# Adult T cell leukemia aggressivenness correlates with loss of both 5-hydroxymethylcytosine and TET2 expression

**DOI:** 10.18632/oncotarget.13665

**Published:** 2016-11-26

**Authors:** Ambroise Marçais, Laetitia Waast, Julie Bruneau, Katia Hanssens, Vahid Asnafi, Philippe Gaulard, Felipe Suarez, Patrice Dubreuil, Antoine Gessain, Olivier Hermine, Claudine Pique

**Affiliations:** ^1^ Service d’Hématologie, Hôpital Universitaire Necker-Enfants Malades, APHP, Université Paris Descartes, Institut Imagine, INSERM U1163-CNRS ERL8254, Sorbonne Paris Cité, Paris, France; ^2^ INSERM U1016, CNRS UMR 8104, Université Paris Descartes, Sorbonne Paris Cité, Institut Cochin, Paris, France; ^3^ Département de Pathologie, Hôpital Universitaire Necker-Enfants Malades, APHP, Université Paris Descartes, Sorbonne Paris Cité, Paris, France; ^4^ INSERM U1068, Centre de Recherche en Cancérologie de Marseille, Institut Paoli-Calmettes, Université de la Méditerranée, Marseille, France; ^5^ Institut Necker-Enfants Malades (INEM), INSERM U1151, and Laboratory of Onco-Hematology, Assistance Publique-Hôpitaux de Paris (AP-HP), Hôpital Universitaire Necker, Paris, France; ^6^ Département de Pathologie, Hôpital Henri Mondor, APHP, Créteil, France; ^7^ INSERM U955, Créteil, France Université Paris Est, Créteil, France; ^8^ Unité d’Épidémiologie et Physiopathologie des Virus Oncogènes, Département de Virologie, CNRS, UMR 3569, Institut Pasteur, Paris, France; ^9^ Imagine Institute, INSERM UMR 1163 and CNRS ERL 8254, Laboratory of Cellular and Molecular Mechanisms of Hemathological Disorders and Therapeutic Implication, Hôpital Necker, Paris, France

**Keywords:** retrovirus, T-cells, leukemia, DNA hydroxymethylation, ten eleven translocation

## Abstract

Mutations in *TET2*, encoding one of the TET members responsible for the conversion of DNA cytosine methylation to hydroxymethylation (5-hmc), have been recently described in Human T-lymphotropic virus type 1-associated adult T-cell leukemia/lymphoma (ATLL). However, neither the amount of genomic 5-hmc in ATLL tumor cells nor TET2 expression has been studied yet. In this study, we analyzed these two parameters as well as the mutational status of *TET2* in ATLL patients. By employing a direct *in situ* approach, we documented that tumor T cells infiltrating lymph nodes exhibit low level of 5-hmc compared to residual normal T cells. Furthermore, this 5-hmc defect was more pronounced in tumor T cells from acute patients than from chronic ones and correlated with reduced expression of TET2 protein. *TET2* variations were found in 14 patients (20%), including 13 with aggressive forms. Strikingly, 9 of the 14 patients showed the same variation (SNP rs72963007), whose frequency in ATLL patients was significantly higher than that of an ethnically matched control population (13% vs. 5%). However, no reduction of 5-hmc was found in PBMC from individuals possessing the variant rs72963007 *TET2* allele, as compared to wild-type individuals. In contrast, a robust correlation was observed between 5-hmc and the levels of *TET2* mRNA. Finally, loss of 5-hmc and TET2 downregulation both correlated with poor survival. These findings demonstrate that ATLL progression coincides with loss of genomic 5-hmc and indicate that downregulation of *TET2*, rather than *TET2* mutations, is the key mechanism involved in 5-hmc modulation during ATLL progression.

## INTRODUCTION

Adult T cell leukemia/lymphoma (ATLL) is a rare and mature T cell malignancy with a very poor prognosis due to Human T-lymphotropic virus type 1 (HTLV-1) infection [1, 2]. Four subtypes have been described: two indolent forms (smoldering and chronic) and two aggressive forms (acute and lymphoma) that are resistant to most conventional therapies with an overall survival below one year [3, 4].

HTLV-1 encodes for two oncoproteins, Tax and HBZ, which both play a key role all along HTLV-1-mediated T-cell transformation. The current model assumes that Tax, through its ability to trigger permanent T-cell proliferation and escape from apoptosis, is the main actor for initial T-cell lymphomagenesis and T-cell immortalization. On the other hand, HBZ, the only viral product with sustained expression in ATLL tumor cells, is believed to be involved in the proliferation and/or survival of the transformed clone (reviewed in [5, 8]). In addition, secondary events such as appearance of genetic and epigenetic alterations are believed to contribute as well to the generation of the malignant clone (reviewed in [9, 10]). Notably, hypermethylation of the viral promoter or cellular genes has been described in ATLL cells, with a positive correlation between the degree of abnormalities and the aggressiveness of the disease [11, 12].

In addition to genomic 5-methylation, which role in regulating gene expression has been well established, genomic 5-hydroxymethylation has more recently emerged as another epigenetic mark *per se* [13]. Conversion of 5-methylcytosine (5mc) to 5-hydroxymethylcytosine (5-hmc) is catalyzed by members of the Ten-Eleven translocation (TET) family, comprising TET1, 2 and 3 (reviewed in [14]). TET2 and TET3 are expressed in CD4+ T lymphocytes while expression of TET1 is very low in these cells [15]. Mutations in the *TET2* gene have been found in a variety of myeloid disorders and in mature lymphoid malignancies [16–20] and have also been recently described in Japanese ATLL patients [21–23]. In mice, TET2 inactivation induces expansion of hematopoietic progenitor cells and pleiotropic abnormalities affecting both myeloid and lymphoid lineages and *Tet2*^−*/*−^ mice develop diverse hematopoietic malignancies with long latency [24–26]. Noteworthy, heterozygous and homozygous *Tet*2-deficient hematopoietic stem cells exhibit similar properties and *Tet2^+/−^* mice also develop hematopoietic malignancies, indicating haplo-insufficiency properties of *TET2* [24–26]. *TET2* mutations in humans are also mostly heterozygous and can occur early in the stem cell compartment or later during lymphoid development [25].

In this study, we examined for the first time the level of genomic 5-hmc and its link with TET2 mutation or expression in a cohort of ATLL patients suffering from either chronic or aggressive ATLL. We provide direct evidence that ATLL tumor cells display low global 5-hmc level as compared to normal T cells and that levels of 5-hmc and TET2 further distinguish tumor T cells from acute patients from those of chronic patients. We also identified TET2 mutations especially in patients with aggressive ATLL but show that these mutations did not impact global 5-hmc level. These findings reveal on one hand that reduced genomic 5-hmc is a marker of ATLL aggressiveness and on the other hand that *TET2* downregulation rather than *TET2* mutations is the main mechanism for 5-hmc loss in ATLL tumor cells.

## RESULTS

### *In situ* detection of 5-hmc and TET2 in ATLL tumor T cells

**Figure 1:**
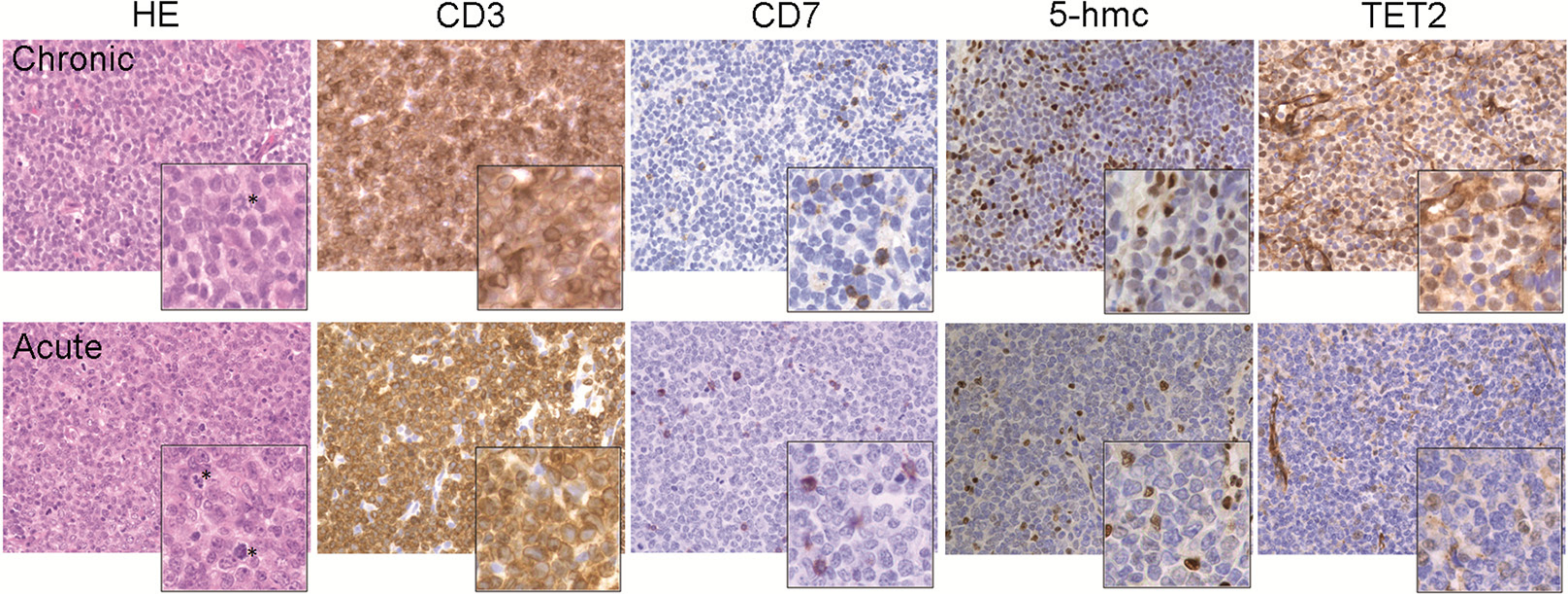
Assessment of 5-hmc level and TET2 expression in ATLL tumor T cells Distribution of 5-hmC and TET2 in lymph node sections from ATLL patients detected by immunohistochemistry. Biopsies were colored with hematoxylin-eosin (HE) and stained with CD3 and CD7 to discriminate tumor T cells (CD3^+/DIM^ CD7^−^) from residual normal T cells (CD3^+^ CD7^+^) as well as with the anti-5-hmc or anti-TET2 antibody. Positive staining appears as brown signal. Magnification ×400 (enlarged image ×2).

No data are available to date regarding the level of genomic hydroxymethylation in ATLL tumors cells. To address this issue, immunohistochemistry (IHC) experiments were performed on lymph node biopsies from an acute or a chronic ATLL patient (Figure 1). As previously reported [27], variation in expression of the CD3 and CD7 T-cell markers can be used to discriminate normal T cells (CD3 high/CD7+) from tumor T cells (CD3dim/CD7-). Tissue sections were therefore stained for CD3 and CD7 as well as for genomic 5-hmc. Examination of the sections showed massive diffuse tissue infiltration by medium to large sized CD3dim CD7- T cells, comprising cells in mitosis (Figure 1, HE; see asterisks), in good agreement with a transformed phenotype. Residual smaller normal T cells strongly expressing CD3 and CD7 could also be detected in the lymph nodes. For each biopsy, strong 5-hmc staining was found in residual normal T cells while poor signal was observed in the predominant population of CD7- tumor cells. Interestingly, levels of 5-hmc appeared higher in tumor cells from the chronic patient as compared to tumor T cells from the acute patient (Figure 1, 5-hmc).

Since TET2 is the main regulator of 5-hmc in hematological cancers [25], TET2 expression was also studied in lymph node biopsies (Figure 1, TET2). While the difference was less pronounced than for 5-hmc, lower TET2 staining was found as well in tumor T cells as compared to normal T cells, suggesting that TET2 accounts, at least in part, for the variation in 5-hmc found between these two populations. Moreover, comparison between tumor T cells from the acute or chronic patient revealed that the TET2 staining mirrored the 5-hmc signals. Indeed, TET2 protein expression was barely detectable in tumor T cells from the acute patient while a significant TET2 signal was observed for the chronic patient.

These data demonstrate that, as compared to normal T cells, ATLL tumor T cells exhibit impaired genomic 5-hydroxymethylation. Moreover, they suggest that a distinct 5-hmc/TET2 pattern may distinguish tumor T cells from acute patients from those from chronic patients.

### Quantification of 5-hmc level and TET2 expression in acute versus chronic patients

**Figure 2:**
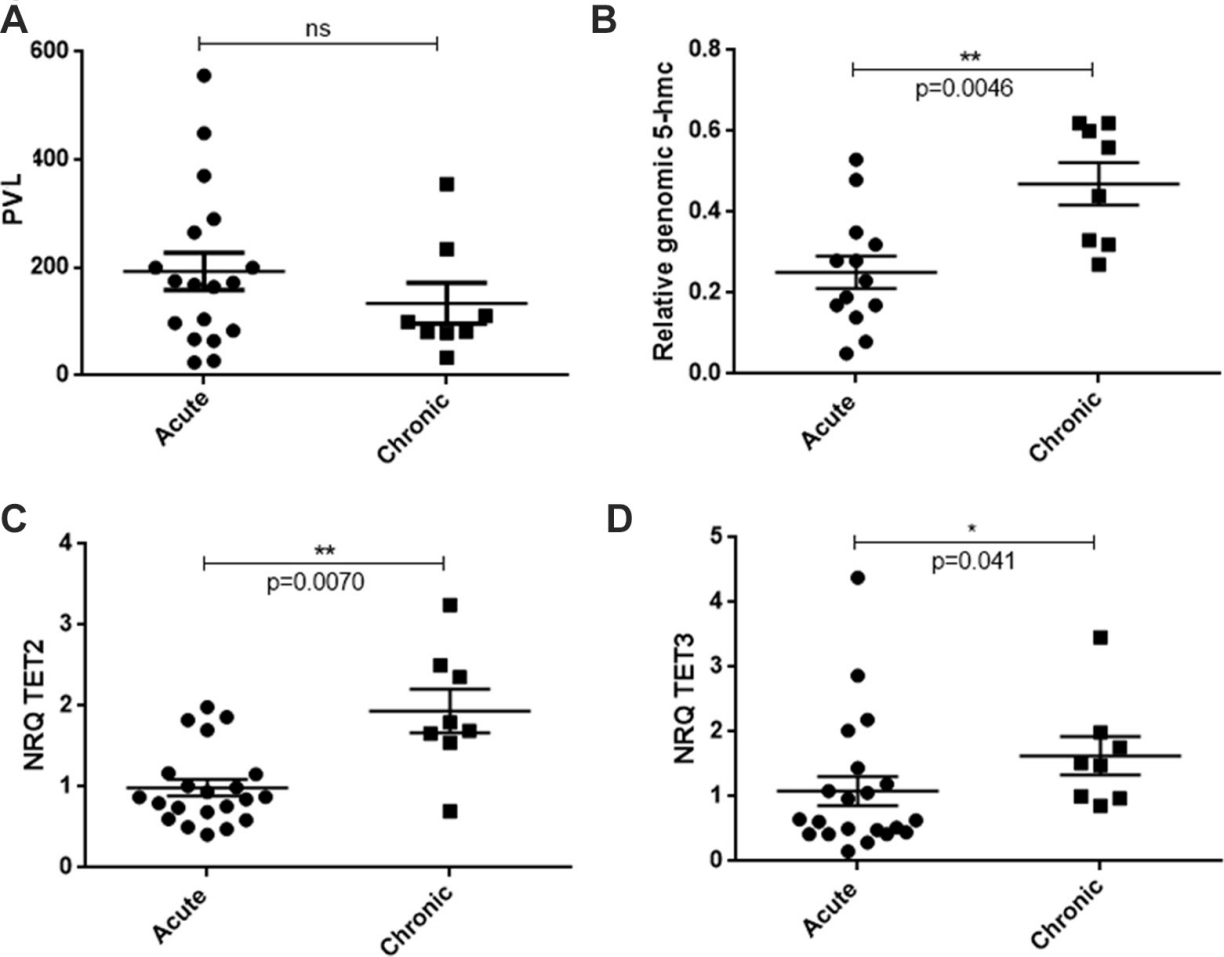
Levels of genomic 5-hmc and TET transcripts in acute versus chronic ATLL patients (**A**) Comparison of proviral loads (PVL) between samples from acute (*n* = 18) or chronic (*n* = 8) patients. (**B**) Statistical analysis (Mann-Whitney test) comparing 5-hmc levels between PBMC from acute (*n* = 13) or chronic (*n* = 8) patients. (**C**, **E**). Statistical analysis (Mann-Whitney test) comparing the level of TET2 (**C**) or TET3 (**D**) transcript in PBMC from acute (*n* = 21) or chronic (*n* = 8) ATLL patients. Bars represent mean values with standard error of the means. Patient details are presented in Supplementary Table S1.

To confirm the above finding, 5-hmc level and TET2 expression were quantified in PBMC from acute versus chronic patients by dot-blot experiments and RT-qPCR, respectively (Figure 2). Since epigenetic marks vary according to cell types, we normalized the contribution of residual normal blood cells in 5-hmc detection. Therefore, only samples containing more than 70% of tumor cells were considered (see Supplementary Table S1). Moreover, no significant difference in proviral load was found between the selected samples from the two groups (Figure 2A), allowing direct comparison. A statistically significant reduction in 5-hmc was found in PBMC from acute patients (*n* = 13) in comparison to PBMC from chronic patients (*n* = 8) (*p* = 0.0046) (Figure 2B). In addition, a 2-fold reduction in *TET2* mRNA was observed in PBMC from acute patients (*n* = 21) as compared to chronic patients (*n* = 8), and this difference was associated to a robust *p*-value (*p* = 0.0070, Figure 2C). Decreased TET3 mRNA expression was also observed but with a *p*-value on the border of statistical significance (*p* = 0.041, Figure 2D). TET1 mRNA expression was almost undetectable in PBMC from either HTLV-1 carriers or ATLL patients, while it was readily detected in tonsil B cells as described [28] (Supplementary Table S2), confirming that TET1 is barely expressed in PBMC [15].

These data confirm that genomic 5-hmc is reduced in acute versus chronic patients, which coincides with a highly significant downregulation of *TET2* mRNA.

### *TET2* genetic variations in acute ATLL patients

**Figure 3:**
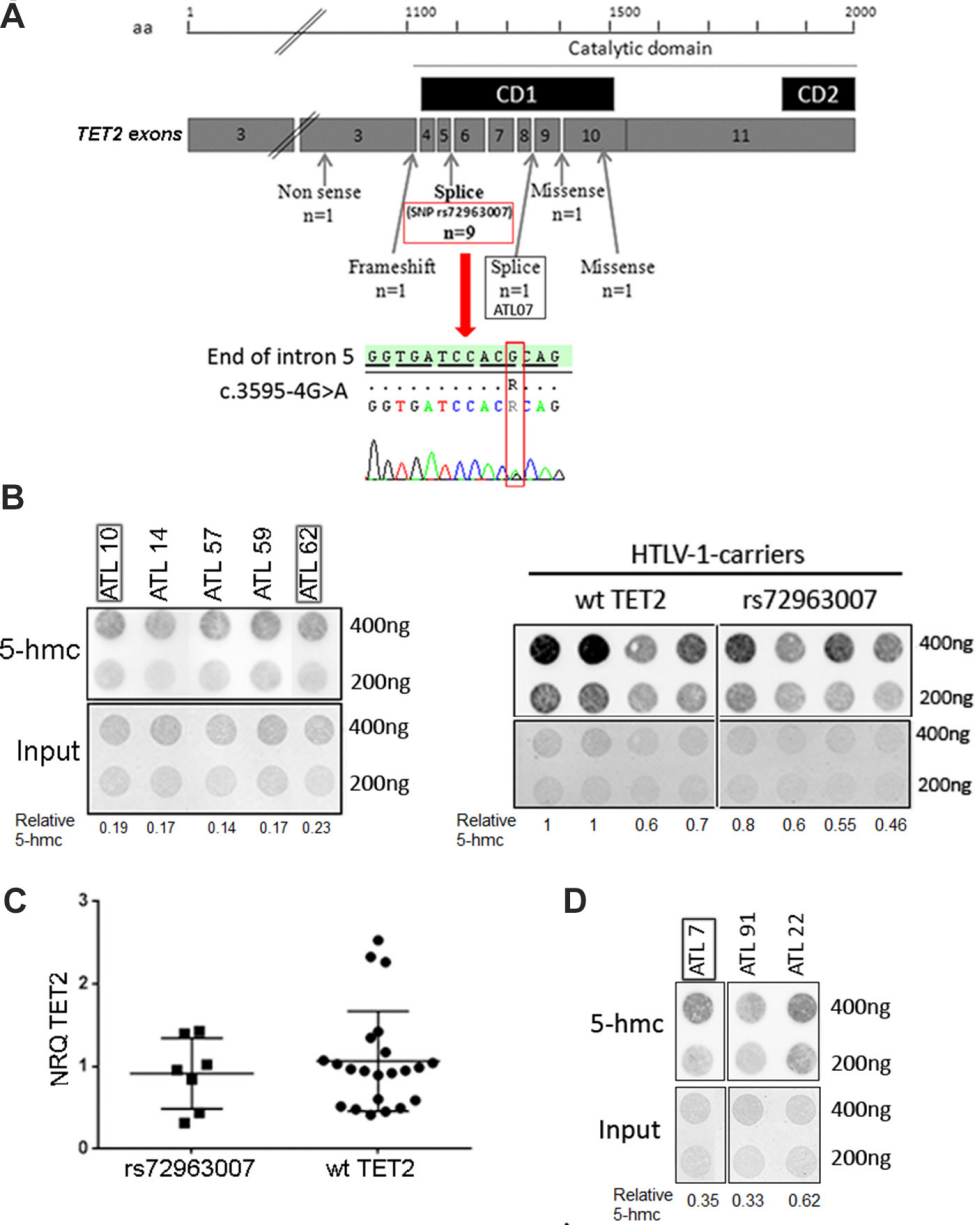
*TET2* mutations in ATLL patients and effect on genomic 5-hmc (**A**) Position and nature of the *TET2* mutations found in ATLL patients. The catalytic domain of TET2 including the two conserved domains (black boxes) are represented. The lower part shows the presence of the rs72963007 variation in one ATLL patient (ATLL 13). (**B**) Detection of genomic 5-hmc in PBMC from acute ATLL patients possessing (black frame, *n* = 2) or not (*n* = 3) the *TET2* rs72963007 variation (left panel) or in PBMC from HTLV-1-infected carriers possessing or not the rs72963007 variation (right panel). (**C**) Levels of *TET2* transcript in PBMC from acute ATLL patients possessing (*n* = 7) or not (*n* = 22) the *TET2* rs72963007 variation. Bars represent mean values with standard error of the means. (**D**) Detection of genomic 5-hmc in PBMC from one acute ATLL patients possessing the *TET2* c.4045–1G > A mutation (black frame) as compared to two wt *TET2* acute patients. Patient details are presented in Table 1 and Supplementary Table S1.

Recent studies have reported the presence of *TET2* mutations in ATLL patients from Japan [21–23]. However, whether *TET2* mutations also exist in ATLL patients from other origins and whether they are specific for a clinical subtype of ATLL have not been investigated yet. To explore these issues, sequencing of *TET2* was performed in a cohort of 71 ATLL patients from African origin and suffering from aggressive (acute and lymphoma) or indolent (chronic and smoldering) form of the disease (Table 1). *TET2* mutations were found in 14 out of 71 patients (20%), including 13/60 patients with an aggressive subtype (acute+lymphoma) and 1/11 patient with indolent subtype (chronic+smoldering) (Table 2 and Figure 3A). Interestingly, although classified as smoldering according to the Shimoyama classification [3], the latter patient (ATLL 06) had a poor outcome and rapidly progressed to a lymphoma subtype.

**Table 1:** Details of the ATLL patients included in TET2 sequencing analysis

Name^a^	Sex	Age	Subtype	Sample type	TET2 status
**ATLL 01**	Female	39	Acute	Blood	Wild-type
**ATLL 02**	Female	51	Chronic	Blood	Wild-type
**ATLL 03**	Female	33	Acute	Blood	Wild-type
**ATLL 04**	Male	31	Chronic	Blood	Wild-type
**ATLL 05**	Female	34	Acute	Blood	Wild-type
**ATLL 06**	Male	51	Chronic	Blood	**rs72963007 (Intron 5)**
**ATLL 07**	Female	59	Acute	Blood	**Splice mutation (Intron 8)**
**ATLL 08**	Male	58	Chronic	Blood	Wild-type
**ATLL 09**	Male	50	Chronic	Blood	Wild-type
**ATLL 10**	Male	48	Acute	Blood	**rs72963007 (Intron 5)**
**ATLL 11**	Male	27	Chronic	Blood	Wild-type
**ATLL 13**	Male	37	Lymphoma	Blood Lymph node	**rs72963007 (Intron 5)**
**ATLL 14**	Female	35	Acute	Blood	Wild-type
**ATLL 15**	Female	62	Acute	Blood	Wild-type
**ATLL 16**	Male	50	Acute	Blood	Wild-type
**ATLL 17**	Male	45	Lymphoma	Blood	Wild-type
**ATLL 18**	Male	56	Acute	Blood	Wild-type
**ATLL 19**	Male	63	Acute	Blood	Frameshift, p.Glu984Asnfs*23 (Exon 3)
**ATLL 21**	Female	69	Acute	Blood	Wild-type
**ATLL 22**	Female	69	Chronic	Blood	Wild-type
**ATLL 23**	Female	41	Chronic	Blood	Wild-type
**ATLL 24**	Female	42	Acute	Blood	Wild-type
**ATLL 25**	Male	53	Acute	Blood	Wild-type
**ATLL 27**	Female	46	Acute	Blood	Wild-type
**ATLL 28**	Female	54	Acute	Blood	**Missense, p.Gln1414Lys (Exon 10)**
**ATLL 29**	Female	50	Acute	Blood	Wild-type
**ATLL 30**	Male	64	Acute	Blood	**rs72963007 (Intron 5)**
**ATLL 31**	Female	45	Lymphoma	Blood	Wild-type
**ATLL 33**	Female	30	lymphoma	Blood	Wild-type
**ATLL 38**	Male	39	Acute	Blood	Wild-type
**ATLL 40**	Female	46	Acute	Blood	**rs72963007 (Intron 5)**
**ATLL 41**	Male	65	Acute	Blood	Wild-type
**ATLL 42**	Female	68	Lymphoma	Blood Lymph node	Wild-type
**ATLL 43**	Female	65	Lymphoma	Blood Lymph node	Wild-type
**ATLL 44**	Female	54	Lymphoma	Blood Lymph node	**rs72963007(Intron 5)**
**ATLL 45**	Female	51	Acute	Blood	Wild-type
**ATLL 46**	Female	46	Lymphoma	Lymph node	**p.Arg1383Gly Missense (Exon 10)**
**ATLL 47**	Male	49	Lymphoma	Lymph node	Wild-type
**ATLL 48**	Female	64	Lymphoma	Lymph node	Wild-type
**ATLL 49**	Male	34	Lymphoma	Lymph node	Wild-type
**ATLL 50**	Male	47	Acute	Lymph node	p.Glu337* Nonsense (Exon 3)
**ATLL 51**	Male	35	Lymphoma	Lymph node	Wild-type
**ATLL 52**	Female	49	Acute	Lymph node	Wild-type
**ATLL 53**	Male	63	Acute	Lymph node	Wild-type
**ATLL 54**	Male	70	Lymphoma	Blood	Wild-type
**ATLL 55**	Male	47	Lymphoma	Blood	**rs72963007 (Intron 5)**
**ATLL 56**	Female	42	Chronic	Blood	Wild-type
**ATLL 57**	Male	47	Acute	Blood	Wild-type
**ATLL 58**	Female	28	Acute	Blood	Wild-type
**ATLL 59**	Male		Acute	Blood	Wild-type
**ATLL 60**	Male	42	Acute	Blood	Wild-type
**ATLL 61**	Female	47	Acute	Blood	Wild-type
**ATLL 62**	Male	64	Acute	Blood	**rs72963007 (Intron 5)**
**ATLL 63**	Female	45	Smoldering	Blood	Wild-type
**ATLL 64**	Male	63	Lymphoma	Blood	Wild-type
**ATLL 65**	Male	54	Lymphoma	Blood	Wild-type
**ATLL 66**	Male	61	Acute	Blood	Wild-type
**ATLL 67**	Male	39	Smoldering	Blood	Wild-type
**ATLL 68**	Female	53	Acute	Blood	Wild-type
**ATLL 69**	Female	62	Acute	Blood	Wild-type
**ATLL 70**	Male	66	Lymphoma	Blood	Wild-type
**ATLL 71**	Male	66	Acute	Blood	Wild-type
**ATLL 72**	Female	34	Acute	Blood	Wild-type
**ATLL 73**	Female	57	Lymphoma	Blood	**rs72963007 (Intron 5)**
**ATLL 74**	Female	61	Acute	Blood	Wild-type
**ATLL 75**	Male	34	Acute	Blood	Wild-type
**ATLL 76**	Female	33	Lymphoma	Blood	Wild-type
**ATLL 77**	Male	68	Lymphoma	Blood	Wild-type
**ATLL 80**	Female	44	Acute	Blood	Wild-type
**ATLL 81**	Male	61	Acute	Blood	Wild-type
**ATLL 82**	Male	40	Acute	Blood	Wild-type

^a^All patients were from African origin and were included at diagnosis. Except ATL 11 who received chemotherapy, all patients received no prior treatment.

**Table 2:** Frequency of *TET2* mutations in ATLL patients

	ATLLCohort (*n* = 71)	%	Aggressive subtype	%	Chronic subtype	%
**Total patients with TET2 sequence variants**	14	20	13	22	1	8
**Patients with TET2 somatic mutations**	5	7	5	8	0	0
**Patients with SNP rs72963007**	9	13	8	13	1	8

Five patients (7% of the cohort), all with aggressive forms, had a specific *TET2* mutation (Figure 3A and Table 1). Further analysis was only possible for 2 patients for which additional PBMC were available. For ATL 07 (acute form), who presented a splice mutation at position c.4045-1 (Table 1), cell sorting was performed to separate the tumor cell population from normal blood cells (see Materials and Methods). The mutation was found in the CD3^dim^/CD4^+^ population, corresponding to tumor T cells, but not in the CD3^high^/CD4^+^; CD3^high^/CD8^+^ and CD14^+^ normal cell populations. For ATLL 19 (acute form), who presented a nucleotide deletion, sequencing was performed from PBMC obtained both at diagnosis and after complete remission and the *TET2* deletion found at diagnosis was no more detected at remission. Therefore, at least in these two patients, the identified *TET2* mutation is specific to the tumor cell population.

Strikingly, among the 14 ATLL patients with a *TET2* mutation, 9 had a recurrent mono allelic nucleotide variation at position c.3595-4G > A (Chr.4: 106164642-106164867) (Table 1). This variation, known as SNP rs72963007, is located 2 nucleotides upstream of the splice acceptor site of *TET2* intron 5 (Figure 3A, lower part). To determine whether rs72963007 was a somatic or a germinal mutation, the mutated region was sequenced from PBMC of three patients with a lymphoma subtype. As expected, no tumor T cells were found in the blood, as demonstrated by the absence of clonal TCR rearrangement and flow cytometry analysis. In each case, the rs72963007 mutation was found in normal blood cells, strongly arguing for its germinal origin.

The *TET2* rs72963007 variation is not uniformly represented worldwide and its frequency varies with the ethnic origin. Frequency reaches ∼ 5% in the African population but is less than 1% in the worldwide population (1000 genomes database). We then measured the frequency of the rs72963007 variation by sequencing the mutated region in 143 HTLV-1-infected carriers ethnically matched with the ATLL cohort. The frequency of rs72963007 in this control carrier population was 5%, similar to that of the African population. A statistically significant higher frequency was found when ATLL patients (aggressive and chronic forms) were compared to carriers (13% vs. 5% *p* = 0,0416, OR = 2,820; 95% CI, 1,004 to 7,921) (Table 3). This observation suggests that the *TET2* rs72963007 variation is a risk factor for developing ATLL, at least in the African population.

**Table 3:** Frequency of the rs72963007 *TET2* SNP in ATLL patients and ethnically-matched HTLV-1 carriers

	HTLV-1 Carriers	ATLL patients (aggressive + chronic)
**Number of patients**	143	71
**Number of patients with the rs72963007 mutation**	7	9
**rs72963007 frequency**	5%	13%
**Odd Ratio (95% CI)**		2.820(1.004 to 7.921)
***p*-value**		0.0416

### Link between 5-hmc and *TET2* mutation or expression and impact on survival

The impact of harboring the rs72963007 *TET2* allele or a *TET2* somatic mutation on genomic hydroxymethylation and TET2 expression in ATLL tumor T cells was next studied. No significant difference in genomic 5-hmc was found between PBMC from acute ATLL patients possessing the *TET2* rs72963007 allele and PBMC from wild-type (wt) *TET2* acute ATLL patients (Figure 3B, left panel). A similar result was obtained when the experiment was conducted with PBMC from HTLV-1-infected carriers possessing or not the variant allele (Figure 3B, right panel). Moreover, PBMC from patients possessing the variant *TET2* allele produced comparable level of *TET2* transcript than wt patients (Figure 3C). Similar levels of 5-hmc were also found between PBMC from patients with the mutated c.4045-1G > A allele and wild-type patients (Figure 3D). These results are in agreement with a previous study showing that *TET2* mutations do not always lead to detectable change in global genomic 5-hmc [29].

**Figure 4:**
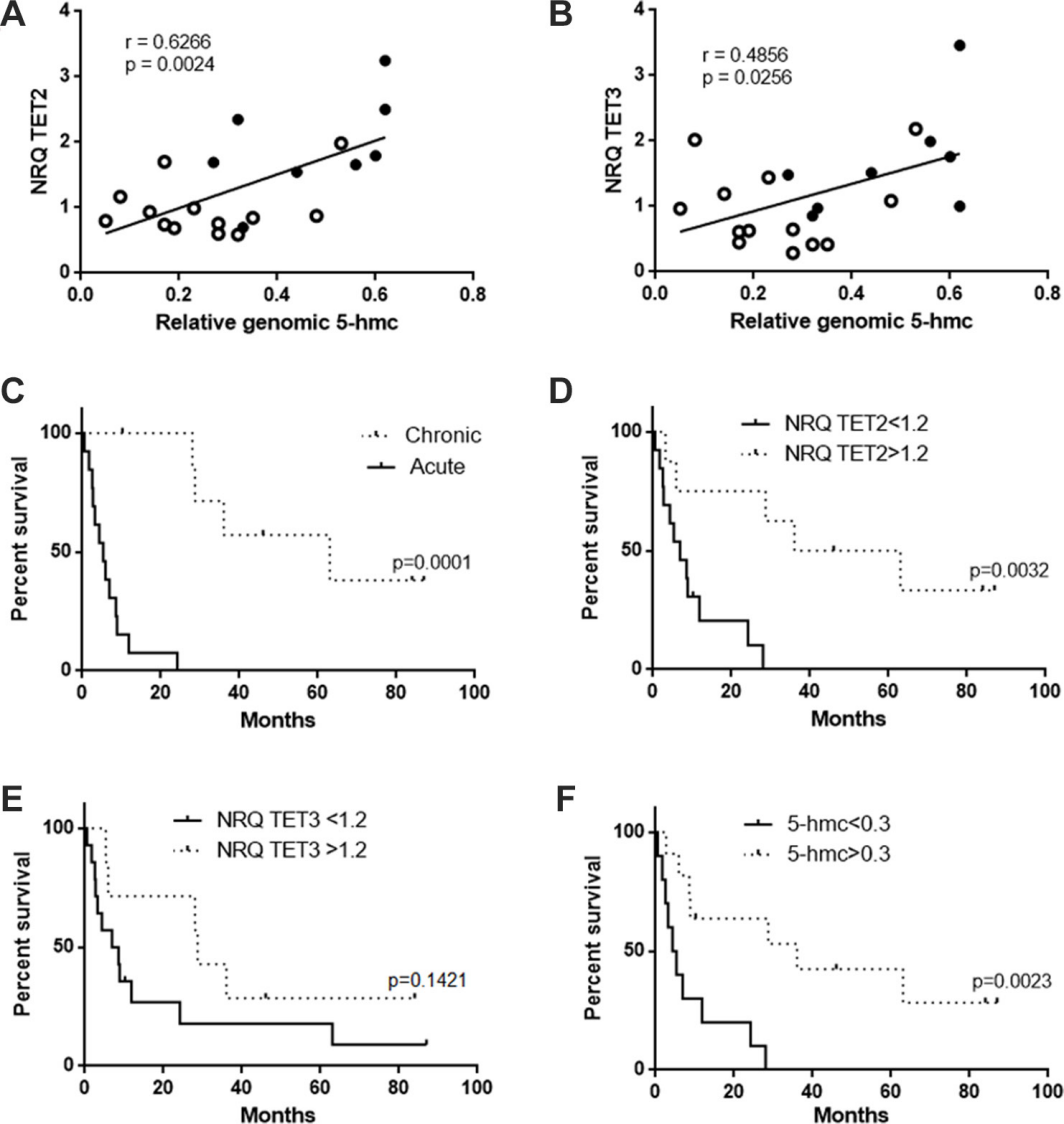
Correlation between 5-hmc level and TET expression and impact on survival (**A**, **B**) Correlation between the level of *TET2* (A) or *TET3* (B) transcript and the level of genomic 5-hmc. Both acute (open circle) and chronic (closed circle) patients were considered for the analysis. (**C**) Survival of acute and chronic ATLL patients. Survival probabilities were estimated by the log-rank (Mantel-Cox) test. Median survival was 5.3 and 63 months for acute and chronic patients, respectively. (**D**) Survival of ATLL patients (acute and chronic) according to the level of *TET2* transcript. The threshold of 1.2 corresponds to the mean TET2 expression of the selected patients. Median survival was 6.9 and 49.5 months for acute and chronic patients, respectively. (**E**) Survival of ATLL patients (acute and chronic) according to the level of *TET3* transcript. The threshold of 1.2 corresponds to the mean TET3 expression of the selected patients. Median survival was 7.75 and 28.7 months for acute and chronic patients, respectively. (**F**) Survival of ATLL patients (acute and chronic) according to the level of 5-hmc. The threshold of 0.3 corresponds to the mean 5-hmc values of the selected patients. Median survival was 4.8 and 36 months for acute and chronic patients, respectively. Patient details are presented in Supplementary Table S1.

In contrast to what we observed for *TET2* mutations, we found a significant correlation between the level of *TET2* transcript and the amount of global 5-hmc for both acute and chronic patients (Figure 4A; *r* = 0.63, *p* = 0.0024) acute patients being essentially distributed among the low TET2/5-hmc values (Figure 4A, open circle) and chronic patients (closed circle) among the high ones. A weaker correlation with 5-hmc was found in the case of TET3 transcript (Figure 4B; *r* = 0.4856, *p* = 0.0256) transcript, confirming the main role of TET2 in 5-hmc modulation.

We finally questioned whether the levels of TET mRNA and 5-hmc have an impact on patient survival. As previously reported [4], survival was significantly lower in acute patients than in chronic patients (*p* = 0.0001; Figure 4C). Survival plot analysis revealed that both low expression of TET2 (Figure 4D) and low genomic 5-hmc (Figure 4F) also strongly correlated with poor survival (*p* = 0.0032 and *p* = 0.0023, respectively). In contrast, no significant difference in survival was found in the case of *TET3* transcript expression (Figure 4E). Hence, both low *TET2* mRNA expression and genomic 5-hmc represent markers of poor prognosis in the case of ATLL.

## DISCUSSION

Genomic 5-hmc is now considered as a stable epigenetic mark often deregulated in cancers [13]. 5-hmc reduction has been observed in solid tumors and hematological malignancies such as chronic myelomonocytic leukemia (CMML), acute myeloid leukemia (AML) and secondary AML (sAML) [30, 31]. Our study provides the first demonstration that reduced 5-hmc is also a hallmark of ATLL tumor T cells. Indeed, using a direct *in situ* approach, we found that tumor T cells infiltrating lymph nodes display low level of 5-hmc while strong 5-hmc signals were observed in residual normal T cells. In addition, we documented a further 5-hmc reduction in tumor T cells from acute ATLL patients in comparison to chronic patients. This demonstrates that as previously reported for increased genomic methylation [11], reduction in genomic hydroxymethylation also correlates with the aggressiveness of ATLL.

A simple explanation could be that reduced 5-hmc is due to *TET2* mutations. Indeed, recent studies reported *TET2* somatic mutations in ATLL patients from Japan (∼10% of patients) [21–23]. Our present data show on the one hand that *TET2* mutations also exist in ATLL patients from African origin and on the other hand, that they are especially detected in the aggressive subtype. Interestingly, one of the somatic *TET2* mutations we found (c.4045-1G > A; ATL07) was previously identified in myelodysplastic syndromes [31]. Moreover, we found that the frequency of rs72963007, a germinal *TET2* variation classified as a single nucleotide variation, was elevated in ATLL patients as compared to ethnically-matched HTLV-1-infected carriers. Of note, neither the rs72963007 mutation nor any other recurrent *TET2* mutations was described in Japanese ATLL patients [21–23]. It remains to investigate whether this is due to a difference in the frequency of rs72963007 between Japanese and African populations. Another recent study reports high *TET2* mutation frequency in ATLL, occurring in 32% (10/31) of ATLL samples from a Japanese cohort [22]. However, it should be stressed that mutations located outside the catalytic domains of TET2 were taken into consideration in this study, rendering difficult to conclude on their impact on TET2 function.

Recent publications have shown that SNPs in cancer-related genes may predispose to lymphoid and myeloid disorders. Two studies recently described the association of two SNPs in the *GATA3* locus with susceptibility, development and prognosis of acute lymphoblastic leukemia [32, 33]. Regarding *TET2*, a study reported the association of the rs4698934 *TET2* SNP with melanoma development whereas somatic mutations are rare in this disease [34]. Interestingly, the rs4698934 is, as the rs72963007 described here, located in an intronic region, [34]. Two other *TET2* SNPs, rs2454206 and rs3733609, have been shown to be either a prognosis marker in pediatric acute myeloblastic leukemia or a predisposition factor in myeloproliferative neoplasm harboring JAK2V617F mutation, respectively [35, 36]. The prognostic impact of *TET2* mutations was not apparent in our series, possibly owing to the extremely poor survival of ATLL. Indeed, the median survival for aggressive forms does not exceed 10 months. We observed that *TET2* mutations were essentially restricted to patients with aggressive subtype of ATLL, except one patient who harbored an indolent subtype but rapidly progressed to an aggressive form.

Little is known regarding the role of genetic predisposing factors in ATLL development. A large Japanese prospective study has shown that one of the major risk factor for developing ATLL is a family history of ATLL [37]. In this study, which includes 1218 HTLV-1 infected patients, the family history of ATLL was an independent risk factor for developing an ATLL with and hazard ratio of 12.1 in the multivariate analysis, suggesting the implication role of genetic predisposing factors. One study has shown that specific HLA alleles predispose to ATLL development in the Japanese population. However, no predisposing SNP in cancer related genes has been identified to date for ATLL [38]. Our results indicate that the rs72963007 *TET2* SNP can be considered as a predisposition factor for developing ATLL, notably the aggressive form.

While *TET2* mutations have been described in ATLL patients, their direct impact on genomic 5-hmc in tumor cells was not evaluated. To address this issue, we performed DNA dot-blot experiments using fresh PBMC from ATLL patients or HTLV-1-infected carriers. We found that neither the rs72963007 nor the c.4045-1G > A *TET2* variation resulted in 5-hmc reduction in ATLL tumor cells. These findings confirm that, even if they are found in patients, some TET2 mutations do not have a major impact on global genomic 5-hmc [29]. However, it could not be excluded that these *TET2* mutations impact methylation of a restricted set of genes. Alternatively, mutations could prevent TET2 interactions with cellular partners also involved in the transformation process. Further investigations will be required to understand mechanisms linking *TET2* mutations to ATLL development and aggressiveness.

*TET2* mutations are only found in a minority of acute ATLL patients [21–23] while as shown here, all acute ATLL patients show reduced genomic 5-hmc as compared to chronic patients. Importantly, we provide an explanation for this observation by showing that this 5-hmc reduction coincides with the downregulation of *TET2*. Indeed, immunohistochemistry experiments allowed us to document that tumor T cells infiltrating lymph nodes from an acute patient express lower amount of TET2 protein than tumor T cells from a chronic patient. Furthermore, we showed that whatever their *TET2* mutational status, tumor T cells from acute ATLL patients express less *TET2* transcript than tumor cells from chronic patients and that, when all patients were considered, there is a strong positive correlation between the level of 5-hmc and the amount of *TET2* transcript. In addition, we showed that both reduced TET2 expression and low genomic 5-hmc correlate with poor survival while survival is not affected by level of *TET3*. These results strongly suggest that the decrease in 5-hmc found in acute ATLL patients is the direct consequence of TET2 downregulation. Taken together, these findings indicate that the main determinant for reduced 5-hmc in ATLL cells is not the appearance of inactivating *TET2* mutations but rather the downregulation of TET2 transcript. In some tumor models, TET2 expression has been shown to be repressed via hypermethylation of CpG islands located in its genomic sequence [39]. Our preliminary data indicate no difference in the degree of methylation of the *TET2* CpG islands between acute and chronic patients (data not shown). Hence, the mechanism involved in TET2 downregulation in acute ATLL patients remains to be elucidated. It would also be of great interest to compare how 5-hydroxymethylation affects the whole genome in tumor cells from acute or chronic ATLL patients.

In conclusion, this study provides the first demonstration that defect in genomic hydroxymethylation, mainly due to TET2 downregulation, contributes to ATLL progression.

## MATERIALS AND METHODS

### Patients and donors

This study was approved by the ethics committee (CPP Ile de France II) and all living patients gave written informed consent. Patients’ details and clinical features are summarized in Table 1 and Supplementary Table S1. Clinical data were collected retrospectively. Samples were obtained at diagnosis and, with the exception of ATLL-11 who received chemotherapy; all patients received no prior treatment (Table 1). Depending on the patients, samples at different time points of the disease or samples from different tissues (blood and lymph node) collected at the same time point were analyzed (Table 1). HTLV-1 carriers were all asymptomatic and were from French Guyana, French West Indies or West Africa, matching the ethnic origins of the ATLL patients. Tonsil B cells, used a positive control for *TET1* mRNA expression [28], were kindly provided by Dr. Y. Richard (Institut Cochin, Paris).

### Isolation of PBMC, cytometry and sorting

Peripheral blood mononuclear cells (PBMC) were obtained by centrifugation in Ficoll gradient and were immediately frozen after purification. The proportion of tumor cells was determined by cytometry analysis by counting the % of CD3^dim^, CD4+, CD25+, HLA-DR+ and CD7- T lymphocytes.

For ATLL 07, cell sorting was performed after staining of PBMC with the following fluorescent primary antibodies: anti-CD3-PE, anti-CD4-Cy7, anti-CD8-APC and anti-CD14-FITC. Four populations were sorted: CD3^high^/CD4^+^; CD3^high^/CD8^+^; CD14^+^; and CD3^low^/CD4^+^ (tumor T cells).

### Gene sequencing

DNA extraction was performed using the Blood DNA Qiagen extraction kit. TET2 gene sequencing was performed as previously described [16–20]. Variations in the entire *TET2* gene sequence that correspond to frameshift, nonsense or splice site mutations were considered. Missense mutations were considered only if they were located within the two conserved regions of TET2 (ranging from amino acids 1104 to 1478 and 1845 to 2002, respectively) [20]. Categorical variables were compared between different groups by the Chi2-test using the GraphPad Prism 6 software.

### DNA dot blot

Dot blot assay was performed as follows. Briefly, genomic DNA samples were diluted in SSC buffer, denatured at 95°C for 10 minutes after addition of NaOH 1,6 M and then loaded on a Hybond N+ nylon membrane (GE Health) using a 96-well dot-blot apparatus. After baking at 80°C for 10 min, the membrane was incubated overnight in blocking buffer containing 5% BSA and 5% non-fat milk, and then incubated for 2 hours at room temperature with the anti-5-hmc rabbit polyclonal antibody (Active Motif #39769, 1:10.000) diluted in blocking buffer. Finally, the membrane was washed in PBS-Tween 0,1% and incubated with an HRP-conjugated rabbit secondary antibody in blocking buffer (1:5000, Promega, France). After incubation with the ECL plus detection kit (ThermoScientific), chemoluminescent signal was captured using a Fusion FX imager (Vilber Lourmat). To control sample loading, the membrane was stained with methylene blue post-immunoblotting. The signals for 5-hmc and input DNA were quantified using Image J and the levels of 5-hmc were normalized to input DNA. Images were processed using the Adobe Photoshop software.

To allow comparison between different experiments, DNA extracted from two PBMC from non-infected donors were included in each dot-blot and only experiments in which less than 20% of 5-hmc variation was found for these samples were selected. A value of 1 was given for the 5-hmc level of one the control PBMC and 5-hmc level of samples from ATLL patients was calculated relative to this value (relative genomic 5-hmc).

### RNA extraction and RT-qPCR

RNAs were isolated from thawed PBMC with the “all PREP DNA/RNA mini kit” (Qiagen) according to the manufacturer's instructions. Reverse transcription was performed with the “Maxima first strand cDNA synthesis kit” (Thermo scientific) according to the manufacturer's instructions. TET2 mRNA expression was detected using the Roche Real time Ready assay ID 144583 and TET1 mRNA expression was detected using the following primers: forward primer AAGGGAGCCAACAAAAATGT/ reverse primer AGGACTCTGGGTTCTGAAAA. PCR were conducted using the following conditions: pre-incubation 95° 10 min ×1, amplification ×45: denaturation 95° 10 sec, annealing 60° 30 sec acquisition, extension 72° 1 sec, cooling. Housekeeping genes EEF1G (forward primer AGATGGCCCAGTTTGATGCTAA/reverse primer GCT TCTCTTCCCGTGAACCCT or HPRT (forward primer TGACACTGGCAAAACAATGCA/reverse primer GGTC CTTTTCACCAGCAAGCT were quantified using the SYBR green method using the following conditions: pre-incubation 95° 5 min x1; amplification x45: denaturation 95° 10 sec; annealing 60° 10 sec; extension 72° 10 sec; acquisition, melting curve, cooling. The normalized relative quantity (NRQ) were calculated according to the following formula: $\text{NRQ}=2^{\Delta \text{Ct Sample}-\frac{\Delta \text{Ct ref 1}+\Delta \text{Ct ref 2}}{2}}$ Mean NRQ were calculated from two independent experiments performed in triplicates.

### Immunohistochemistry analysis

Tissues sections were colored with hematoxylin and eosin staining (HE) and immunohistochemistry was performed with the following antibodies: anti-CD3 (Dako, polyclonal, 1/200), anti-CD7 (Novocastra, LP15, 1/100), anti-5-hmc (Active Motif #39769, 1/10.000) and anti-TET2 (Abcam, ab124297, 1/100). All staining were performed with an automated Stainer Leica Biosystem Bond III.

### Quantification of HTLV-1 proviral load

HTLV-1 proviral load was quantified by real-time PCR using primers specific for the pX region (pX-F; 5′-CAAACCGTCAAGCACAGCTT-3′ and pX-R; 5′-TCTCCAAACACGTAGACTGGGT-3′) and the following probe: 5′ 6-FAM-TTCCCAGGGTTTGGA CAGAGTCTTCT-TAMRA-3′. Runs were performed in a 50 μL volume containing 1 μg of total DNA extract, primers and probe (a 200nM concentration of each), 1X PCR buffer (PlatinumQuantitative PCR SuperMix-UDG). Thermocycling conditions were 2 min at 50°C and 10 min at 95°C, followed by 50 cycles at 95°C for 15 sec and 60°C for 1 min. Quantification was standardized with the Tarl2 cell line (HTLV-1, single proviral copy). Albumin was used for normalization. The following primers: forward 5′-GCTGTCATCTCTTGTGGGCTGT-3′ and reverse 5′- AAACTCATGGGAGCTGCTGGTT-3′, respectively and probe: 5′-FAM-CCTGTCATGCCCACACAAATCTC TCC-TAMRA-3′. Results are expressed as HTLV-1 proviral copies per 100 PBMCs.

### Statistical analysis

Statistical analyses were conducted with the GraphPad Prism 6 software. Two-group comparison was performed with the Mann-Whitney test. Survival probabilities were estimated by the Kaplan–Meier method, and pairwise comparisons were made using a log–rank test. Statistical significance was defined for *p*-values < 0.05.

## SUPPLEMENTARY TABLES


