# Supplementary material for: Adult T cell leukemia aggressivenness correlates with loss of both 5-hydroxymethylcytosine and TET2 expression
Source: Oncotarget. 2016 Nov 26;8(32):52256–68. doi: 10.18632/oncotarget.13665 (PMC5581026; doi:10.18632/oncotarget.13665)
Supplement: Supplementary file 1 [file oncotarget-08-52256-s001.pdf]

Adult T cell leukemia aggressivenness correlates with loss of both 5-hydroxymethylcytosine and TET2 expression

Supplementary Materials

Supplementary Table 1: Details of the samples included in the 5-hmc and/or RT-qPCR analyses

Name	Subtype	% Tumor cells	PVL[a]	5-hmc	TET2[b]	TET3[b]	Survival[c]
ATLL 01	Acute	85%	27	ND	1,86	4,38	79.6 (1)
ATLL 05	Acute	85%	104	0,05	0,798	0,965	1.7 (1)
ATLL 07	Acute	> 80%	83	0,35	0,845	0,42	8.8 (1)
ATLL 10	Acute	95%	175	0,19	0,6885	0,6295	6.9 (1)
ATLL 14	Acute	85%	265	0,17	0,74	0,61	11.8 (1)
ATLL 25	Acute	95%	64	0,48	0,875	1,085	2.7 (1)
ATLL 28	Acute	96%	24	ND	1,825	0,5	4.6 (1)
ATLL 30	Acute	100%	290	ND	1,155	1,055	7.7 (1)
ATLL 36	Acute	95%	172	0,53	1,985	2,185	5.9 (1)
ATLL 38	Acute	90%	200	ND	1,01	2,87	5.6 (0)
ATLL 57	Acute	85%	67	0,14	0,935	1,19	24.2 (1)
ATLL 59	Acute	>60%	164	0,17	1,7	0,45	3.2 (1)
ATLL 61	Acute	90%	97	0,28	0,76	0,29	4.3 (1)
ATLL 62	Acute	90%	99	0,23	0,99	1.44	2.5 (1)
ATLL 66	Acute	97%	449	ND	0,87	0,52	1.3 (1)
ATLL 71	Acute	95%	556	ND	0,48	0,48	4.5 (1)
ATLL 74	Acute	98%	ND	0,28	0,6	0,65	0.5 (1)
ATLL 75	Acute	70%	168	0,08	1.17	2.02	5.3 (1)
ATLL 80	Acute	73%	ND	ND	0,5	0,42	21 (1)
ATLL 82	Acute	71%	>200%	ND	0,41	0,15	9.8 (1)
ATLL 88	Acute	98%	370	0,32	0,59	0,42	8.6 (1)
ATLL 02	Chronic	85%	355	0,27	1,69	1,48	28 (1)
ATLL 08	Chronic	77%	79	0,32	2,355	0,855	87 (0)
ATLL 11	Chronic	80%	33	0,62	3,25	3,46	84 (0)
ATLL 22	Chronic	>70%	110	0,62	2,5	1	63 (1)
ATLL 23	Chronic	80%	81	0,6	1,795	1,76	28.7 (1)
ATLL 37	Chronic	75%	80	0,44	1,545	1,515	36 (1)
ATLL 56	Chronic	70%	99	0,56	1,66	1,99	46 (0)
ATLL 91	Chronic	83%	234	0,33	0,7	0,97	10.2 (0)

[a]Proviral load is expressed as number of proviral copies per 100 PBMC.
[b]Mean Normalized Relative Quantity (NRQ) were calculated from two independent experiments performed in triplicates.
[c]Survival is expressed in months. Number 1 or 0 indicates death or survival of the patients, respectively.
ND: not determined.

Supplementary Table 2: Comparison of TET, TET2 or TET3 mRNA expression in PBMC from carriers or ATLL patients

	2-ΔCp Tet1 /HPRT	2-ΔCp Tet2 /HPRT	2-ΔCp Tet3 /HPRT
**Carriers HC5**	8,62E–04	3,67E–01	1,71E–01
**ATLL 37**	1,16E–03	5,30E–01	1,82E–01
**ATLL 61**	1,12E–03	2,32E–01	5,98E–02
**Tonsil B cells**	3,91E–02	ND	ND

ND: not determined.
